# Internalization mechanisms of cell-penetrating peptides

**DOI:** 10.3762/bjnano.11.10

**Published:** 2020-01-09

**Authors:** Ivana Ruseska, Andreas Zimmer

**Affiliations:** 1 Institute of Pharmaceutical Sciences, Department of Pharmaceutical Technology and Biopharmacy, University of Graz, 8010 Graz, Austria

**Keywords:** cell-penetrating peptides, direct translocation, drug delivery, endocytosis, internalization

## Abstract

In today’s modern era of medicine, macromolecular compounds such as proteins, peptides and nucleic acids are dethroning small molecules as leading therapeutics. Given their immense potential, they are highly sought after. However, their application is limited mostly due to their poor in vivo stability, limited cellular uptake and insufficient target specificity. Cell-penetrating peptides (CPPs) represent a major breakthrough for the transport of macromolecules. They have been shown to successfully deliver proteins, peptides, siRNAs and pDNA in different cell types. In general, CPPs are basic peptides with a positive charge at physiological pH. They are able to translocate membranes and gain entry to the cell interior. Nevertheless, the mechanism they use to enter cells still remains an unsolved piece of the puzzle. Endocytosis and direct penetration have been suggested as the two major mechanisms used for internalization, however, it is not all black and white in the nanoworld. Studies have shown that several CPPs are able to induce and shift between different uptake mechanisms depending on their concentration, cargo or the cell line used. This review will focus on the major internalization pathways CPPs exploit, their characteristics and regulation, as well as some of the factors that influence the cellular uptake mechanism.

## Introduction

The cell membrane is a semipermeable barrier, serving as a protective layer for the cells. It is an essential organelle for cell survival and function. As a barrier, it only allows the transport of compounds with small molecular size, which can be transported using channels and specific carriers [1–2]. Macromolecules, however, are unable to use these modes of entry [2].

The production of novel therapeutic molecules, which do not adhere to the “canonical” rules defining what a drug molecule should be like, has been accelerated these days. One part of this new group are proteins, peptides and nucleic acids, all developed with one thing in mind – bypassing the limitations of conventional therapeutics [3]. The novelty of these macromolecular compounds lies in their ability to target specific molecules and biological pathways, and thus, modulate molecular activities in a positive or negative manner [4]. The outstanding possibilities offered by the use of such molecules (proteins, peptides and nucleic acids) in the diagnostics and treatment of a number of diseases render them exceptionally attractive. Nonetheless, there are some obstacles that need to be overcome, such as the limited cellular uptake and low target specificity of these molecules. In order to do so, we are in great need for new delivery and administration strategies.

Thus far, a plethora of cellular translocation techniques have been developed – either using biophysical methods (such as microinjection, electroporation and magnetofection), biochemical methods (for example, the use of amphipathic detergents) and viral vectors [5]. However, no matter how effective, these methods have shown to cause cytotoxic effects, and when it comes to viral vectors, a high probability of viral gene insertion into the host genome exists [6]. Therefore, the delivery strategy for macromolecules is still left as an unanswered question. In the best case scenario, an efficient delivery system would provide enzymatic protection and stability for the drug, an improved distribution and target specificity, as well as a lack of toxicity [3].

### Cell-penetrating peptides as drug delivery systems

Having in mind the attention they have gained, cell-penetrating peptides (CPPs) have become a current hotspot in medical research [7]. Compared to the other translocation techniques mentioned, CPPs are capable of entering the cells in a noninvasive manner, they do not destroy the integrity of the cellular membranes and are considered highly efficient and safe. Thus, they provide new avenues for research and applications in life sciences [8]. In general, CPPs can be defined as diverse peptides with a maximal length of 30 amino acids. They are characterized by a high content of basic amino acids and an overall positive charge. CPPs are known to have a high rate of permeation into cells and are able to cross membranes of different cell types, while showing low cytotoxicity and no immunological response [6].

This class of peptides was first introduced in the late 1980s, with the discovery of the TAT peptide, encoded by the human immunodeficiency virus type 1 (HIV-1) by Frankel et al. [9], who showed that the TAT peptide could enter cells and translocate into the nucleus. The following decade unfolded with the discovery of the neuronal cell internalization of penetratin, the first ever CPP. Penetratin was derived from the third helix of the homeodomain of *Drosophila antennapedia*. This discovery was closely followed by the development of two peptides used for the noncovalent delivery of proteins and peptides, MPG and Pep-1 [10]. Today, we have a myriad of CPPs and databases which allow one to browse existing CPPs based on chemical modifications, category, cargo or peptide lengths [6].

### Classification of cell penetrating peptides

CPPs are currently classified in several ways, depending on their individual properties. Table 1 presents a short overview of some of the most commonly used CPPs, listing their amino acid sequence and properties. In this review, only a short outlook of their classification will be given. For more detailed information about the classification of CPPs, reviews are listed in references [5,7,11].

**Table 1:** Overview of some the most commonly used CPPs describing their sequence, class and charge.

Name	Amino acid sequence	CPP class	Charge	Ref.
				
TAT	YGRKKRRQRRR	cationic	8	[9]
penetratin	RQIKIWFQNRRMK WKK	cationic	7	[24]
R9	RRRRRRRRR	cationic	9	[67]
MPG	GALFLGWLGAAGSTMGAPKKKRKV	amphipathic	24	[31]
Pep-1	KETWWETWWTEWSQPKKRKV	amphipathic	2	[30]
transportan-10	AGYLLGKINLKALAALAKKIL-amide	amphipathic	4	[5]
PepFect6	stearyl-AGYLLGK(ε-TMQ)INLKALAALAKKIL	amphipathic	10	[20]
Bac7	RRIRPRPPRLPRPRPRPLPFPRPG	proline-rich	9	[7]

Based on the interaction between the CPP and the therapeutic agent, two main classes of peptides can be distinguished. The first class includes CPPs which form a covalent conjugate with the cargo by chemical cross-linking or by cloning, followed by the expression of a CPP fusion protein. Such interactions have been seen in several CPPs such as TAT derivatives, penetratin or polyarginines [10]. It seems that covalent modification is most suitable for charge-neutral oligonucleotides such as peptide nucleic acids (PNAs) and phosphorodiamidate morpholino oligonucleotides (PMOs) [3,12]. Concerning charged molecules such as siRNA or miRNA, the covalent conjugation leads to restricted biological activity, most likely due to the steric hindrance caused by the covalently-linked CPP, which is an obstacle for the incorporation of siRNA/miRNA into the RISC complex. Because of this, the siRNA/miRNA molecule cannot be loaded into the complex and manifest its effect [13].

Regarding covalent strategies of cargo-attachment, there is a great promise in biorthogonal chemistry, which allows for the design of efficient conjugation reactions even in a complex biological environment [14–16]. Provided that a cleavable covalent bond is achieved, the charged molecule could be released in the cytoplasm and exert its effect. Thus, a bond such as the disulfide one, could be the Holy Grail in covalent CPP-siRNA/miRNA delivery [17]. It has been reported that CPP-siRNA conjugates have reduced transient and stable expression of reporter transgenes in a number of mammalian cell lines [18]. In this case, thiol-containing siRNAs were synthesized and conjugated to penetratin or transportan by a reducible disulfide bond, and there was no change in the structure or activity of the siRNA molecule. Another report for a successful disulfide link between the TAT peptide and siRNA comes from Chiu and co-workers [19]. The stable complex was able to achieve the localization of the siRNA in specific cytoplasmic compartments in the perinuclear region. Andaloussi et al. also developed a stable CPP-siRNA system, derived from transportan-10, containing a proton-acceptor moiety and a stearyl group (for lipophilic interaction), called PepFect6. This system has shown to promote siRNA delivery to difficult-to-transfect cells [20].

The second class within this classification scheme is formed by CPPs which noncovalently complex their cargo. They occur mostly as amphipathic peptides, consisting of a hydrophilic and a hydrophobic domain. Pep-1 and MPG are amphipathic peptides which are reported to form stable, noncovalent complexes with cargo molecules through electrostatic interaction. Pep-1 has successfully been used to deliver small peptides and proteins into cells, while MPG has shown to efficiently deliver small interfering RNA (siRNA) into cultured cell lines [3,10].

The interplay between hydrophilic and hydrophobic amino acids in the sequence of CPPs as well as their length give rise to the second type of classification. According to these properties, CPPs can be regarded as primary or secondary amphipathic or nonamphipathic peptides. Primary amphipathic peptides contain typically more than 20 amino acids and have sequentially hydrophilic and hydrophobic amino acids in their primary structure [5,21]. This group includes MPG, penetratin, CADY, pVec, and other peptides. Secondary amphipathic peptides commonly have less than 20 amino acids in their sequence and are able to take their α-helix or β-sheet conformation after interaction with phospholipid membranes [5]. Secondary α-helical CPPs have a highly hydrophobic patch on one face, whereas the other face can be cationic, anionic or polar. Amphipathic β-sheet CPPs are based on one hydrophobic and one hydrophilic stretch of amino acids exposed to the solvent. A class of secondary amphipathic CPPs are the proline-rich peptides [7]. Nonamphipathic peptides are rather short peptides, such as HIV-TAT, which have a high content of positively charged amino acids such as arginine or lysine. Studies suggest that at least eight positive charges are necessary for efficient uptake to occur [7].

### Cellular uptake of CPPs

Over 30 years of the discovery of CPPs have passed, and their internalization mechanism remains yet to be deciphered. Although their uptake has been reported in a wide variety of cell types and in combination with different cargoes, the exact entry path still remains a question. It is of crucial importance for the overall safety and efficacy assessment that the internalization behavior of CPPs is evaluated. Furthermore, knowledge of the uptake mechanism can be essential for the development of CPP-delivery systems with cell-specificity and low toxicity.

The complexity in resolving this matter arises from the intrinsic properties of the peptides, such as their charge distribution and length. These characteristics allow them to interact with numerous cell surface molecules, which can influence the choice of an entrance path in a great manner [7]. The above mentioned factors, just a few out of many, guide the internalization paths of CPPs to two major routes: endocytosis (active or energy-dependent uptake) and membrane translocation (direct/passive or energy-independent uptake). Overall, the type of uptake which will be selected mainly depends on the physicochemical properties of the peptide and the cargo as well as the concentration applied, in combination with the structural properties of the plasma membrane. As an example, nowadays it is well established that at physiological conditions and low peptide concentration endocytosis prevails, while when a peptide is applied at higher amounts, it translocates the plasma membrane directly. A deviation from this rule is penetratin, which at low concentration passes the membrane in an energy-independent manner, while at higher concentration, it switches to endocytosis [22]. Thus, the challenge which remains, from a therapeutic point of view, is to recognize and point out the uptake route which produces a relevant biological response.

**Figure 1:**
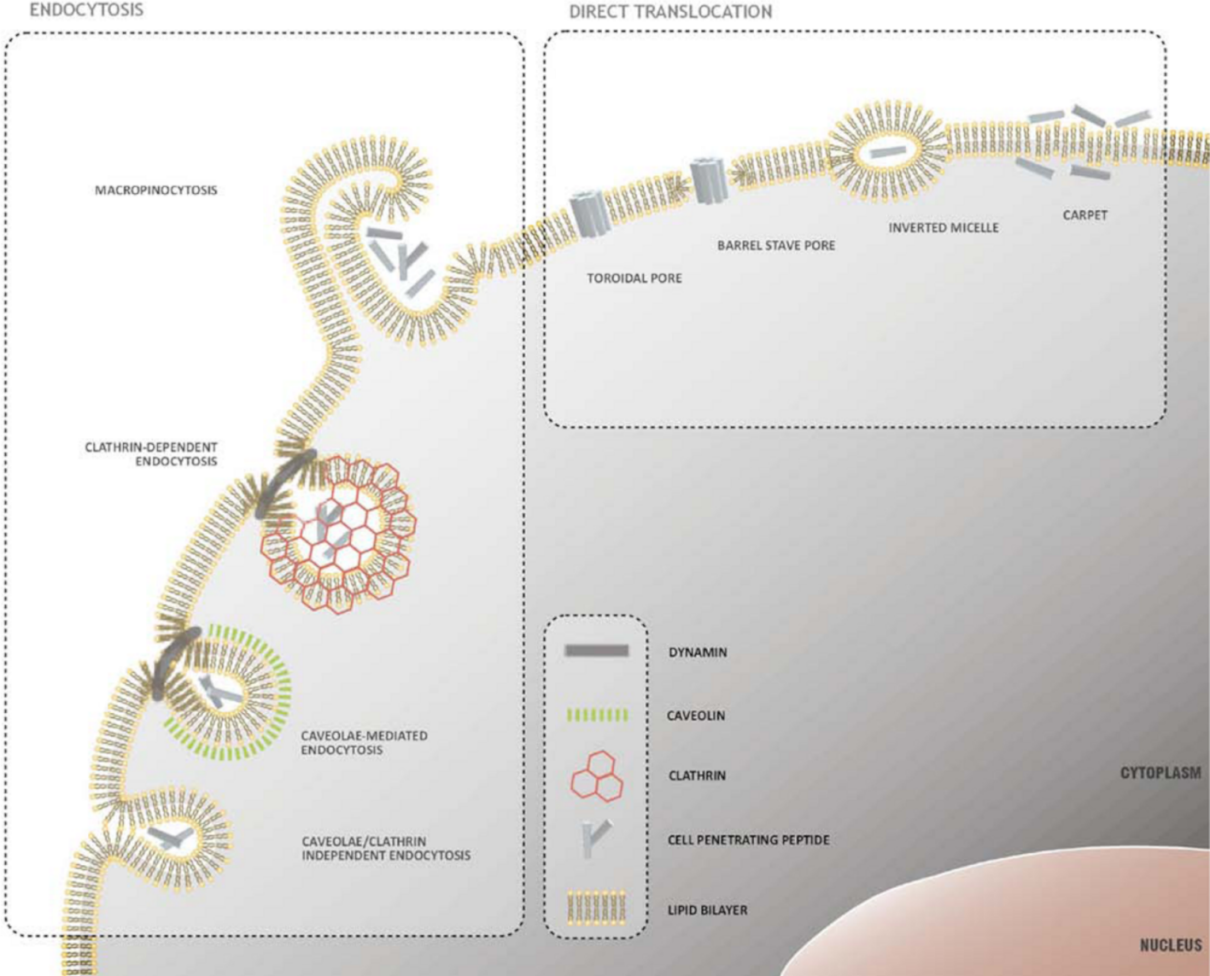
Mechanisms of CPP uptake. Two main mechanisms have been proposed: direct translocation through the cellular membrane, which requires no energy, and endocytosis, an energy-dependent process. Reprinted from [23], copyright Trabulo et al., 2010. CC-BY 3.0 (https://creativecommons.org/licenses/by/3.0/).

Despite the deciphering of the uptake mechanism of CPPs being a work in progress, it is still not a scheme which should be build up from scratch. A “core” consensus exists, according to which the initial step toward CPPs’ uptake is the interaction with cell surface proteoglycans, via electrostatic forces. Additionally, interactions with several membrane proteins have been described as well [7]. The following parts will focus on the major internalization paths CPPs exploit, as well as the parameters which have a major impact on the selection of uptake mechanism. Furthermore, the recent advances in the knowledge of the uptake mechanisms used by the most prominent CPPs are discussed. Figure 1 gives an overview of the active and passive uptake mechanisms CPPs use to enter cells.

## Review

### Direct translocation through the cell membrane

The direct translocation of CPPs through the cell membrane as an energy-independent mechanism and an alternative to endocytosis was suggested after internalization of CPPs was observed at low temperature [23]. As a process which requires no energy, direct translocation is regarded as a single-step process including mechanisms involving the formation of inverted micelles, pores and the ‘carpet’ model [21]. This process can be tested under specific experimental conditions – low temperature, energy depletion and the use of endocytic inhibitors for instance. In general, direct translocation requires the interaction of positively charged CPPs with negatively charged components of the cellular membrane such as the phospholipid bilayer, which then leads to the CPP entrance [6]. Furthermore, direct translocation requires permanent or temporary destabilization of the membrane for internalization to occur. It is generally accepted that direct translocation occurs at high CPP concentration and is most probable for primary amphipathic CPPs such as transportan analogues and MPG [1].

#### Inverted micelle formation

Direct translocation via the formation of inverted micelles was initially reported for penetratin as a mechanism involved at the early stages of cellular uptake [24]. Penetratin is a protein transduction domain derived from the homeoprotein Antennapeadia. It is one of the first peptides described that was able to successfully carry active molecules into cells and is one of the most studied CPPs thus far [25].

The first step in the internalization process is the formation of electrostatic interaction between the peptide and the cellular membrane, which affects the lipid supramolecular organization. This process may lead to changes in the membrane curvature [26]. Such membrane curvatures or invaginations can lead to the formation of inverted micelles that entrap the peptide. The hydrophilic environment inside the inverted micelle allows accumulation of the peptide and is favorable for the transport of hydrophilic compounds conjugated to the peptide. Subsequently, the micelle is destabilized and the peptide–cargo complex is released in the cytoplasm. However, recent data suggest that penetratin internalization occurs via direct translocation or endocytosis depending on the penetratin concentration.

#### Direct translocation via pore formation

In analogy to the inverted micelle formation model, the membrane perturbation observed during internalization of peptides led to the proposition of alternative uptake mechanisms. Direct translocation via pore formation includes two different models, the ‘barrel-stave’ model and the ‘toroidal’ model.

The ‘barrel-stave’ model is characteristic of amphipathic α-helical peptides. These peptides form bundles after interacting with the cellular membrane, which have channels in their centers. The pore is formed by the inwardly facing hydrophilic surfaces and the interaction between the outwardly facing hydrophobic residues with the lipid membrane [27].

On the other hand, the ‘toroidal’ model applies for peptides which are able to form α-helices when they come in touch with cellular membranes. According to this model, the interaction between the positive side chains of the peptide and the phosphate groups leads to the accumulation of the peptide on the outer leaflet of the membrane [28]. The peptides then cause bending of the lipid monolayer into the interior, forming a hydrophilic gap in the membrane, in which phospholipid heads and peptides are found.

The transient pore formation models are in general proposed as mechanisms used by primary amphipathic peptides [1]. The Pep-family of peptides belongs to the group of primary amphipathic peptides. The leader peptide, Pep-1, is a short peptide which efficiently promotes the delivery and cellular localization of a variety of peptides, proteins and antibodies in a broad spectrum of cell lines. It has a length of 21 amino acids and consists of three domains, each conferring a specific function: (i) a hydrophobic, tryptophan-rich sequence required for the interaction with macromolecules and cell-membrane targeting, (ii) a hydrophilic sequence, rich in lysine, derived from the nuclear localization sequence (NLS) of SV-40 large T antigen, necessary for improving the solubility and the intracellular localization of the peptide and (iii) a spacer domain, which improves the flexibility. It has been reported by Deshayes et al. [29] that the internalization of Pep-1 occurs via transient pore formation depending on the formation of α-helices.

**Figure 2:**
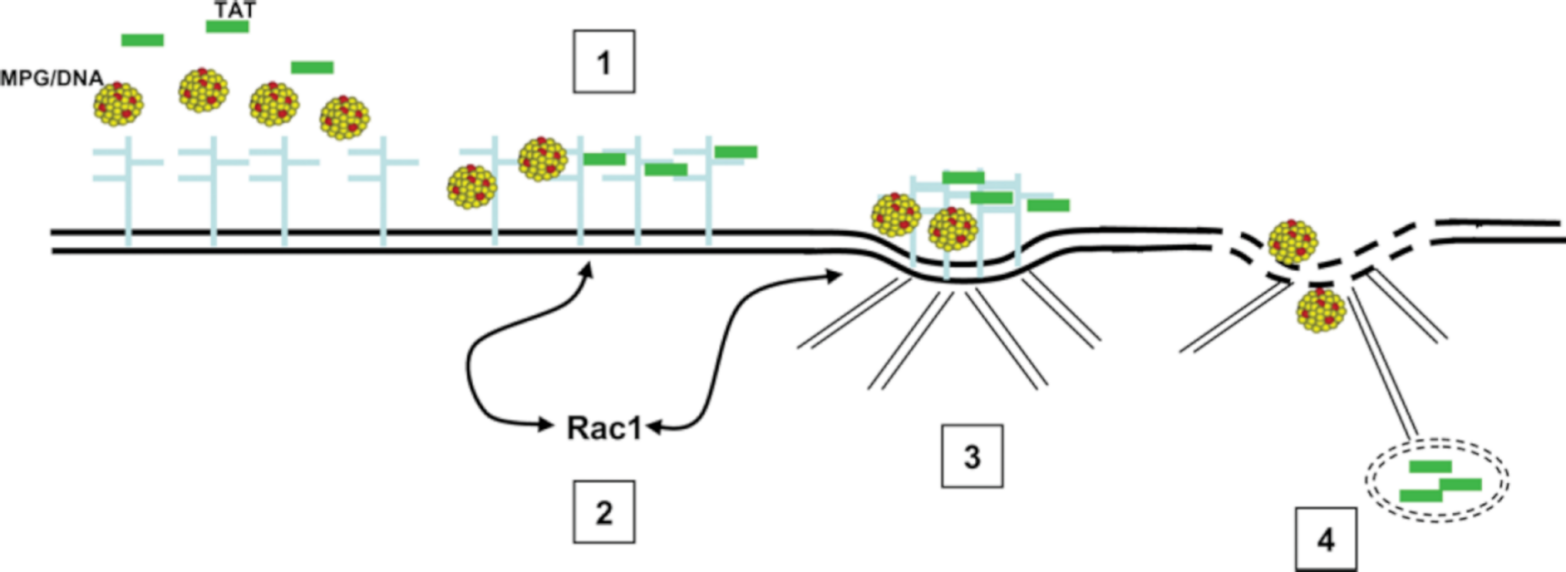
Model for the initial step of cellular uptake of MPG or MPG/cargo complexes. (1) Binding of MPG or MPG/cargo to the extracellular matrix via the proteoglycan platform; (2) clustering of glycosaminoglycans (GAGs), which in turn activates the Rac1 GTPase; (3) actin network remodeling; (4) increase of membrane fluidity, promoting MPG uptake. Reprinted with permission from [32], copyright 2007 The Royal Society of Chemistry.

Pep-1 internalization has been claimed to be independent of the endosomal pathway, which results in limited degradation of the peptide and its cargo inside the cells as well as rapid release of the cargo as soon as the cell membrane has been crossed. It was demonstrated that the free form of Pep-1 interacts strongly with the lipid components in the membrane, leading to a conformational change – the peptide tends to form α-helices. The conformational transitions have been confirmed by CD, NMR and FTIR data. The helical structure that Pep-1 obtains when interacting with the cell membrane favors its insertion into the membrane by forming a transient, transmembrane pore-like structure. Helical folding has also been observed for Pep-1/cargo complexes, suggesting that the cargo does not affect the peptide uptake process [30]. Membrane perturbation as an internalization mechanism has also been proposed for the MPG-family of amphipathic peptides (Figure 2) [3,17,31].

MPG carriers are amphipathic peptides able to form stable complexes with antisense oligonucleotides, plasmid DNA, siRNA and peptides, which improve their stability and cellular uptake. MPG bears three domains: (i) a hydrophobic domain derived from the fusion domain of HIV-1 gp41, (ii) a lysine-rich nuclear localization sequence (NLS) derived from SV-40 large T antigen with five positive charges and (iii) a spacer sequence. Studies performed at low temperature with several inhibitors present, which interfere with the endosomal pathway, have suggested that the uptake of MPG and MPG-cargo complexes does not involve endocytosis. Most likely, MPG internalization is associated with the ability of the peptide to interact with lipids and induce membrane destabilization rather than with active uptake [32].

Studies on the conformational states of MPG and its ability to interact with phospholipids demonstrate that MPG undergoes a transition from unordered into a folded state upon interaction with lipids. What is interesting here is that the new conformational state of MPG is a β-sheet, which differs from the helices that Pep-1 forms, although the two peptides have only slight differences in their structure. Furthermore, MPG folds into β-sheets upon interaction with its cargo, an action that leads to a more pronounced β-sheet folding induced by the phospholipids. This new conformational state eases the formation of transient pore-like structures in the membrane, which leads to the translocation of the MPG/cargo complexes [33].

MPG also shows an inherent ability to induce membrane permeability, whether associated with cargo or not. This process might be due to the actin remodeling allowed by the GTPase Rac 1, a regulatory molecule activated by electrostatic interaction between MPG and GAGs in the extracellular matrix. The two aforementioned actions might constitute the ‘onset’ of the internalization mechanism and have a part in the impact of MPG on membrane fluidity and permeability [32].

#### Carpet model

The ‘carpet’ model describing the direct penetration of some peptides was proposed in 1992 by Pouny and co-workers [34]. According to this model, the positively charged segments of the peptide lie parallel to the membrane surface and are bound to the acidic phospholipid headgroups. The peptides self-associate in a ‘carpet’-like manner. It is postulated that the hydrophobic sites are embedded in the lipid region of the membrane, while the hydrophilic parts orient towards the hydrophilic region, which in turn causes structural reorganization and internalization of the CPP. Since the hydrophobic interaction is necessary for this model, it seems unlikely that it is being used for the internalization of strongly cationic peptides such as TAT. According to this, it is logical that this mechanism was first proposed for dermaseptin, which is an antimicrobial, amphipathic peptide. Electrostatic interaction is essential for the binding between the CPP and the membrane. Achieving a high local concentration at the membrane’s surface is also a key factor for inducing membrane penetration by this model [35].

An alternative to the ‘carpet’ model is the ‘membrane-thinning’ effect, which was first proposed for maganin. Maganin is an amphipathic peptide, composed of 23 residues, which exhibits a broad-spectrum of antimicrobial activity. This model is characterized by a ‘carpet’ formation followed by a perturbation resulting from the interaction of the negatively charged lipids in the outer leaflet in the membrane and the cationic groups of the CPP. This causes a lateral rearrangement of the negative charges and a thinning of the membrane. The aggregation of the CPPs at the membrane surface provokes a reduction of the local surface tension and allows for intercalation of the CPP within the membrane. After the internalization of the peptide, the membrane reseals [36–37].

#### Direct translocation mechanisms used by arginine-rich peptides

The debate regarding the internalization of cationic, arginine-rich peptides, has been long going, and still, the exact mechanism remains to be understood. Initial studies indicated a direct translocation across the cellular membrane that bypassed endocytosis and the involvement of specific receptors. Indeed, cationic CPPs were shown to traverse membranes at low temperature and in the presence of metabolic or endocytic inhibitors. However, in 2003, Richard et al. [38] showed that the results obtained might be a misconception due to the use of fixed cells. They postulated that the fixatives could change the intracellular distribution of the peptides. Additionally, it was shown that flow cytometry (a method frequently used for internalization studies) could not distinguish between peptides that are bound to the membrane and those inside the cell. What is more, experiments using living cells showed that the majority of the CPPs is associated with the outer leaflet of the cell membrane. This evidence led to the conclusion that an energy-dependent process is the major route for the internalization of CPPs.

Nonetheless, novel studies on living cells show that the uptake of arginine-rich peptides could be a combination of both direct translocation and endocytosis. What supports this hypothesis is the mixture of punctate and diffuse staining observed using confocal microscopy [39–40]. It is assumed that the punctate staining indicates endocytic uptake, while the diffuse staining is correlated with direct translocation. The switch between different uptake mechanisms might be concentration-dependent. It has been shown that at low concentration, arginine-rich CPPs are mainly endocytosed, whereas rapid cytoplasmic entry occurred at higher concentration [41]. The latter is associated with the accumulation of the peptide at certain membrane areas called nucleation zones [42]. New findings of these type could further broaden what is already a very wide horizon in the field of CPP research – their internalization mechanism.

The first mechanism explaining the direct penetration of arginine-rich peptides exploits the importance of guanidine groups. This concept was first proposed by Sakai et al. [43], who showed that oligo-arginines could be partitioned into lipid phases from the aqueous phase in the presence of phosphatidylglycerol, a behavior of arginine often referred to as “arginine magic”. The guanidine group found on arginine has proven to form bidentate hydrogen bonds and electrostatic interaction with sulfate, phosphate and carboxylate moieties, all of which can be found on cell surface components. It is thought that the formation of these hydrophobic counterion complexes promotes the accumulation of CPPs on the cell surface and leads to their internalization. However, upon membrane translocation, the peptide backbone has to cross the lipid core. It is assumed that there is hydrophobic interaction between the less hydrophilic peptide backbone and the lipid core involved in this process. The aforementioned translocation processes have been observed for octa-arginine (R8) and the HIV TAT peptide [44].

Studies conducted on the internalization of dodeca-arginine (R12) in HeLa cells, however, suggest a different uptake behavior. Hirose et al. [45] suggest the formation of “particle-like” structures during the interaction and uptake of poly-arginines. The “particles” form simultaneously and have a diameter of 1–3 µm. It is suggested that both membrane components and R12 are involved in the formation of these “particles”. Furthermore, their formation as well as the peptide uptake occur at low temperature (4 °C) in the first 10–20 min of incubation. The authors suggest that the membrane-peptide particle-like structures lead to membrane inversion, which they proved by detecting phosphatidylserine on the cell surface using annexin V. The formation of particle-like aggregates was also reported by Ziegler et al. [46], who investigated the uptake of fluorescently labeled HIV TAT in fibroblasts.

The third mechanism used by the arginine-rich peptides to directly cross the cellular membranes involves the formation of pores. A theoretical model using molecular dynamics simulations was proposed for the translocation of the TAT peptide, which explains the relevance of peptide–phosphate interaction during the pore formation [47]. This theoretical model was later proven experimentally in osteosarcoma and human smooth muscle cells [48]. According to the proposed model, when a concentration threshold is achieved on one of the membrane leaflets, the TAT peptides are attracted to the phosphate groups on the other leaflet. The peptides act cooperatively to facilitate translocation. As a higher TAT concentration is reached, phosphate groups from neighboring phospholipids are drawn to the peptide due to the opposite electrical charge. This process divides the membrane into regions rich in TAT and phosphate groups and into uncharged regions and leads to membrane thinning. TAT forms “complexes” with the phospholipids, due to the interaction between the arginine and lysine side chains with the negatively charged phosphate groups, which then start penetrating the membrane. Simultaneously, water molecules penetrate and solvate the charged groups. With time, the effect of the water molecules results in a transient water pore. Carrying along the attached phospholipids, TAT moves smoothly onto the pore walls and crosses the membrane [47]. Pore formation and translocation can be achieved only after a certain number of peptides is introduced to the membrane surface [49]. A theoretical model for membrane translocation by the formation of water pores has been suggested for hexa-arginine as well [50].

### Endocytosis as an active pathway for CPP uptake

The transport of essential small molecules such as amino acids, sugars and ions occurs through the action of integral membrane protein pumps and channels. Macromolecules, however, require a different machinery in order to traverse the cellular membrane, which usually needs energy. Endocytosis is the active process in which macromolecules are carried into the cell in vesicles or vacuoles pinched-off of the plasma membrane and involves two distinct steps: endocytic uptake followed by endosomal escape [51].

**Figure 3:**
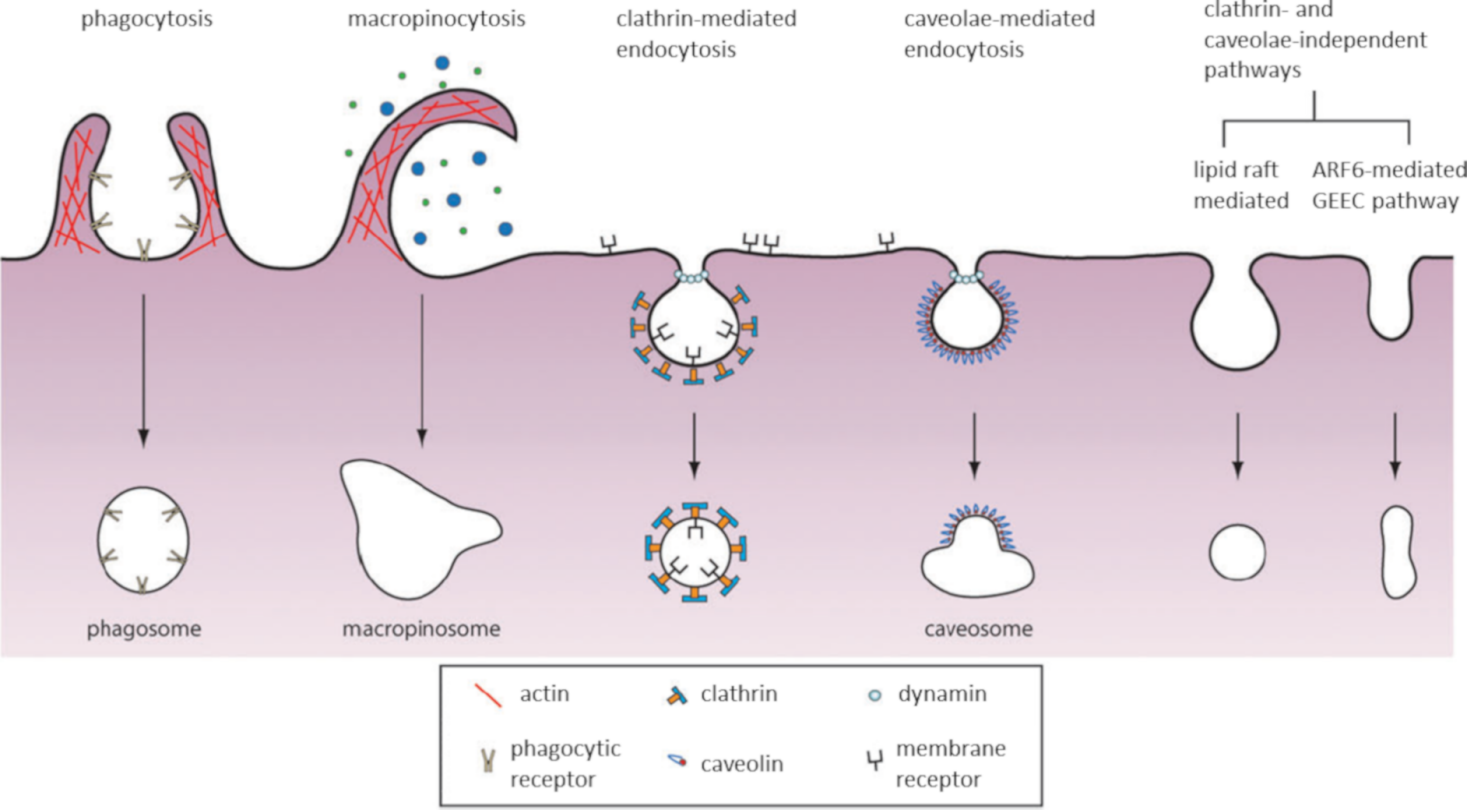
Mechanisms of endocytic entry into the cell. Reprinted with permission from [53], copyright 2011 The Royal Society of Chemistry.

Endocytosis is a complex process composed of more than one mechanism and is generally divided into two categories: phagocytosis and pinocytosis. Phagocytosis involves the uptake of large particles and is restricted to specialized cells (macrophages, monocytes and neutrophils). Pinocytosis, on the other hand, involves the uptake of fluids and solutes and occurs in all cells. At least four different mechanisms have been described for pinocytosis: macropinocytosis, clathrin-mediated endocytosis (CME), caveolae-mediated endocytosis (CvME) and clathrin- and caveolae-independent endocytosis (Figure 3) [52].

All of the endocytic mechanisms described depend on distinct components and mechanisms. To some extent, the choice of pathway can be determined by the cell types and their state of differentiation. However, when it comes to the internalization of nanocarriers such as CPPs, their physicochemical properties and surface reactivates are also important [54].

It is now generally recognized that CPPs at low concentration, and when conjugated to cargo, are taken up by cells in an energy-dependent manner. Endocytosis as a mechanism for the transport of CPPs across cellular membranes was suggested in 2003, after Richard et al. [38] pointed to the possible errors in the results describing direct translocation due to the experimental methods used. Since this initial report, studies which described the active transport of CPPs emerged. Most of the older studies conducted on this matter as well as more recent ones suggest macropinocytosis as the main entry path for CPPs into cells [55–59]. Table 2 summarizes the endocytic mechanisms used by each CPP conjugate mentioned in the following text.

**Table 2:** Summary of the endocytic mechanisms used by CPP–cargo conjugates.

Macropinocytosis	Clathrin-mediated endocytosis (CME)	Caveolae-mediated endocytosis (CvME)	Clathrin- and caveolae-independent endocytosis
			
TAT-protein conjugates [60]	unconjugated TAT [83]	TAT-protein conjugates [92–93]	p18 and p28 azurin fragments [98–99]
poly-arginines [65–67]	MPG/siRNA conjugate [84–85]	proline-rich CPPs [94]	transportan [95]
NickFect51 [77]	NickFect51-cargo conjugates [77]	transportan-10-protein conjugates [95]	low molecular weight protamine (LMWP)/siRNA conjugate [104]
	octa-arginine (R8) [44]	PepFect14/DNA conjugate [97]	
		p18 and p28 azurin fragments [98–99]	
		CVP1 [100]	

#### Macropinocytosis as an entry mechanism

Macropinocytosis is a rapid, lipid raft-dependent and receptor-independent form of endocytosis [60]. It is a process accompanying the membrane ruffling induced in many cell types upon stimulation by growth factors or other signals. Macropinocytosis involves an actin-driven membrane protrusion that results in an increase in fluid-phase uptake [61]. These protrusions do not ‘envelop’ a ligand-coated particle, but instead they collapse onto and fuse with the plasma membrane to generate large endocytic vesicles called macropinosomes [52].

Although macropinocytosis was initially thought to be a nonregulated process, it is now known that this uptake process is a highly organized one. Macropinocytosis consists of quite a few signaling events which involve the remodeling of the cytoskeleton. Most of the macropinocytosis regulators belong to the group of kinases (such as Src, PI3) and GTPases (Rho family, Ras family, Rab proteins), which trigger the actin-driven formation of membrane protrusions [62–64].

As mentioned above, macropinocytosis normally occurs in response to stimulation by growth factors such as the macrophage colony-stimulating factor-1 (CSF-1), the epidermal growth factor (EGF) and the platelet-derived growth factor or tumor-promoting factors [63]. However, this mechanism provides an effective path for drug delivery. It has been described as the pathway used to deliver arginine-rich CPPs such as octa-arginine and TAT peptides into cells.

Kaplan et al. investigated the internalization of the TAT peptide in living cells [56]. The active uptake of TAT was established after experiments were conducted at low temperature, at which all molecular movement in the cell membrane is essentially arrested. After exposing the cells to β-cyclodextrin (which depletes cell surface associated cholesterol) and macropinocytosis-specific inhibitors (such as cytochalasin D, an inhibitor of F-actin or EIPA, an inhibitor or the Na^+^/H^+^ exchange), a dose-dependent reduction of the internalized peptide was observed, which led to the conclusion that TAT transduction into cells occurs by lipid raft mediated endocytosis. Similar results regarding the internalization mechanism of TAT-protein conjugates were reported by Wadia and co-workers [60].

Macropinocytosis has also been suggested as the active uptake process used by other cationic, arginine-rich peptides. Studies conveyed on the internalization of octa-arginine (R8) peptide in HeLa cells showed that the uptake was significantly suppressed by the macropinocytosis inhibitors EIPA and cytochalasin D. In accordance with these results, it was also observed that octa-arginine treatment caused significant rearrangement of the actin cytoskeleton, a process that seems to be crucial for macropinocytosis [55]. Nona-arginine (R9), dodeca-arginine (R12) as well as Flock-House-Virus-derived peptide, also lead to cell internalization via macropinocytosis [65–67]. Furthermore, it is possible that dodeca-arginine acts as a potential target for CXCR4, which is a chemokine receptor that induces macropinocytosis [66].

Recently, a great amount of effort has been put into deciphering the interaction of CPPs with the extracellular matrix as well as with membrane components, which cause the actin rearrangement and lead to the internalization of peptides. The role of glycosaminoglycans in the initial contacts of CPPs and cells and in the initiation of internalization has been a part of several reviews so far [3,27–28,68–69].

The extensive research done on arginine-rich peptides, especially octa-arginine (R8) by Nakase et al. [55], sheds some light on the interaction that leads to macropinocytosis. Their initial studies showed that oligo-arginines as well as TAT provoke actin rearrangement in the initial moments of interaction with cell membranes. The ruffling which was caused by the peptides was similar to the one caused by the interaction of VEGF and its receptor, VEGFR. This led to the suggestion that there are sequence similarities between the basic, arginine-rich domains in CPPs and the growth factors that are known to provoke macropinocytosis. Further research focusing on the importance of the membrane-associated proteoglycans heparan sulfate proteoglycan (HSPG) showed that HSPG was necessary for the uptake of R8, and even more interestingly, it was essential for the uptake of TAT. The observed difference might be due to the higher positive charge of R8 that allows the peptide to interact with more proteoglycans rather than just HSPG. This would suggest that HSPG might be a primary receptor for the cellular uptake of some cationic peptides [61,70].

Syndecans, single transmembrane domain proteins which act as co-receptors for G protein-coupled receptors, have also been proposed to have part in the initiation of macropinocytosis. After the interaction with extracellular ligands, multimerization of syndecans is thought to occur, which in turn induces actin polymerization. Arginine-rich peptides have shown to have this effect on the cellular signal transduction via syndecan multimerization. Multimerization of syndecan-4 was observed in the presence of R8, followed by an increase in the internalized amount of the peptide [71–72]. All of the aforementioned interactions lead to the internalization of arginine-rich peptides by the activation of Rac protein.

On the other hand, Pang et al. [73] propose a different set of interactions that could induce the macropinocytosis of CPPs. In this case, the research group investigated the uptake of TAT-peptide functionalized nanoparticles and they proposed the CendR (C-end-rule) pathway as a possible mechanism that activates macropinocytosis. This route involves the NRP1, a transmembrane protein and a co-receptor of various ligands, and it has several structural requirements for the peptide: it should have C-terminal arginine with a free α-carboxyl group and the natural ʟ-conformation. It is thought that the binding of TAT to NRP1 could induce macropinocytosis independent of HSPG. However, it seems that NRP1 and HSPG work simultaneously in the induction of the active uptake of CPPs. Furthermore, the interaction with this protein might be specific for CPPs conjugated to nanoparticles and perhaps macromolecules.

Since the membrane-bound proteoglycans have been associated with the activation of membrane ruffling and macropinocytosis, it seems logical to conclude that their interaction with arginine-rich peptides will induce the aforementioned path. However, more evidence is needed in order to state that proteoglycans act as receptors for macropinocytosis. Tanaka et al. [66] demonstrated that the uptake of dodeka-arginine (R12) can be mediated by the CXCR4 chemokine receptor. This conclusion is supported by the inhibition of R12 internalization by a CXCR4 knockdown as well as the colocalization of R12 and CXCR4 observed by LSCM in macropinosomes in cells. R8 and TAT did not activate the CXCR4-mediated uptake, which might explain why there is a higher cellular uptake efficiency of R12 over these peptides. Further findings on the internalization of R8 suggest lanthionine synthetase component C-like protein 1 (LanCL1) as a possible receptor that could provoke R8 macropinocytosis. LanCL1 is considered a cytosolic, peripheral membrane protein, which was found to stimulate R8 uptake in HeLa cells. The exact biological function of the protein is not yet clearly defined. Therefore, further studies are needed to address in detail the contribution of LanCL1 in the promotion of R8 uptake [74].

Scavenger receptors are a family of cell surface glycoproteins first recognized to bind modified low-density lipoproteins (LDL) such as acetylated and oxidized LDLs. These receptors have been reported to mediate the translocation of negatively charged CPP/cargo complexes through cellular membranes [75]. Scavenger receptors are known to bind promiscuously to polyanionic ligands and were shown to be involved in multiple endocytic pathways (macropinocytosis, CME, and caveolae-dependent endocytosis). In several papers, it has been demonstrated that the scavenger receptors class A3 and 5 (SCARA3 and SCARA5) are at least partially responsible for the uptake of CPPs [76]. Arukuusk et al. [77] have reported the involvement of SCARA3 and SCARA5 in the uptake of the anionic CPP NickFect51, a stearylated transportan 10 (TP10) analog, via macropinocytosis.

#### Clathrin-mediated endocytosis (CME)

The advanced understanding of the molecular mechanisms governing clathrin-mediated endocytosis (CME) makes this uptake mechanism the best-characterized type of endocytosis thus far. CME is a receptor-dependent, clathrin-mediated and dynamin-required process [78]. Clathrin-mediated endocytosis occurs in all mammalian cells and supports the continuous uptake of essential nutrients such as LDL particles, which carry cholesterol to cells and bind to the LDL receptor (LDLR), and iron-laden transferrin (Tfn) that binds to Tfn receptors (TfnR) [52]. It is a crucial process throughout the life of an organism, as it is responsible for the uptake of transmembrane receptors and transporters, for remodeling the plasma membrane composition in response to environmental changes and for regulating cell surface signaling.

Clathrin-mediated endocytosis can be generally described as a process involving the strong binding of a ligand to a specific cell surface receptor, resulting in the assembly of clathrins in a polyhedral lattice on the cytosolic surface of the cell membrane. This process is followed by the invagination of the clathrin-coated membrane surface towards the cytoplasm and formation of a coated pit that adopts the shape of a spherical membrane structure with a diameter of 100–150 nm. Shallow pits undergo progressive invagination into dome-like shapes, which are connected to the plasma membrane by a funnel-like rim. Further invagination leads to the formation of a spherical bud, and the rim transforms into an hourglass-like membrane neck. Eventually, the neck undergoes fission [79]. For this step, dynamin, a kind of GTPase, is required. In subsequent steps, the released clathrin-coated vesicles (CCVs) are rapidly uncoated and delivered to early endosomes, which mature to late endosomes. Late endosomes then deliver their cargo to lysosomes, organelles characterized by a very low pH value, which is usually the last step in this uptake process [39,78]. Clathrin-mediated endocytosis has been proposed as another mechanism that arginine-rich CPPs use for their uptake.

**Clathrin-mediated endocytosis as a highly organized process:** Early studies on clathrin-mediated endocytosis focused on nutrient receptors which are constitutively internalized (such as the TfnR and LDLR). This led to CME being understood as a constitutive process. In their review on the regulation of CME, Mettlen et al. [80] compare this viewpoint on CME with the circulation of buses according to a set timetable irrespective of the number and destinations of passengers. This analogy is quite correct, having in mind that CCVs were thought to form at a fixed rate, independent of the cargo. Nowadays, data show that CME is a highly regulated and cargo-driven process.

CME can be divided into five stages: (i) initiation of endocytic events, (ii) cargo loading, (iii) membrane bending, (iv) vesicle scission and (v) disassembly of the coat. Each of the stages mentioned is highly orchestrated by a series of molecular interactions [80].

The initiation phase, the first step in this biochemical pathway, is the focal point for regulation. It defines the site where the endocytic vesicle will be formed and is likely the key stage for regulating the frequency of endocytic events. The so-called hot spots for vesicle formation are usually zones in the plasma membrane enriched with phosphatidylinositol 4,5-biphosphate (PI(4,5)P_2_) which interacts with adaptor proteins. However, local differences in the concentration of cargo could also provoke recruitment and clustering of adaptor proteins. The pioneer molecules which initiate the formation of the CCV are adaptor proteins, working together with scaffold proteins. The most important adaptor protein is adaptor protein 2 (AP2) complex, which binds to PI(4,5)P_2_ and then recruits scaffold proteins to the plasma membrane. Due to allosterically regulated AP2 conformational changes caused by the PI(4,5)P_2_ and cargo binding, as well the scaffold proteins, AP2 triggers clathrin assembly [80–81].

The second step in CME is called “cargo loading”. Basically, it involves the binding of clathrin-coat components to the cytosolic regions of the transmembrane cargo molecules. This drives the clustering of all cargo molecules to one region of the membrane, where the clathrin-coated vesicle will be formed. Oftentimes this point in CME has been referred to as “cargo checkpoint”, meaning that if a certain threshold of cargo molecules is not reached, the process of vesicle budding will be either delayed or aborted [82].

Membrane bending is the following step in CME. Several endocytic modules contribute to the formation of a membrane curvature: the coat, the actin filaments, and the scission proteins. Clathrin is the coat-component that has a part in the membrane bending process. When it binds to the adaptor protein complex on the plasma membrane, clathrin rapidly assembles into icosahedral cages. It is thought that the polymerization of clathrin could be responsible for the membrane curvature. The actin cytoskeleton also contributes to membrane bending during CME. There is evidence that rapid actin polymerization occurs in the region surrounding the coat and the base of the growing membrane invagination. After vesicle scission, actin filaments depolymerize in seconds [79,82].

Vesicle scission is the process where the CCV is separated from the donor membrane. This step is catalyzed by the large GTPase dynamin. First, dynamin assembles into tight oligomers, allowing constriction of the membrane neck. After GTP hydrolysis, dynamin oligomers further constrict in the presence of GTP. The constricted state of the membrane causes spontaneous transitions to a hemi-fission and then to a fission state [82]. Recent evidence also suggests that dynamin is necessary in the initial steps of vesicle formation [80].

The disassembly of the coat is the process in which the new vesicle is released to fuse with an early endosome. In addition, the endocytic machinery proteins are also released so that they can be reused for another endocytic event. This process is promoted by an ATPase activity, which leads to clathrin and dynamin depolymerization. Dephosphorylation of PI(4,5)P_2_ to phosphoinositol 4-phosphate also mediates the coat disassembly [80,82].

**Clathrin-mediated endocytosis and CPPs:** A common trait of all CPPs is their ability to switch between different uptake mechanisms depending on multiple exogenous factors. Clathrin-mediated endocytosis has been described as one mechanism CPPs can use to traverse cell membranes. Thus far, it has been reported as a pathway used by the TAT peptide, oligo-arginines as well as by anionic CPPs.

After denoting endocytosis as an uptake mechanism for CPPs, the work of Richard et al. [83] continued to focus on the specific endocytic paths used by the peptides. In 2005 they suggested that the uptake of full-size TAT peptides in HeLa cells occurs through clathrin-mediated endocytosis. Their findings were supported by the use of specific CME inhibitors, which led to a decrease in the uptake of the peptide. After uptake, TAT was targeted to acidic compartments in the cytoplasm, which is in accordance with the CME flow. They speculated that the interaction between TAT and heparan sulfates plays a significant part in the internalization, although these receptors are not a prerequisite for TAT entry. However, this implies only for unconjugated TAT, as it has been demonstrated that there is an alternative uptake pathway for TAT in the presence of conjugated cargo. Around the same time, it was reported that MPGα/siRNA complexes were also taken up by CME, although earlier MPG had shown to enter cells in an energy-independent manner [84–85]. MPGα is a derivative of the original MPG peptide. It is possible that the type of attached cargo influences the chosen uptake pathway, since it was shown that CME inhibitors decreased the amount of MPGα/siRNA complexes inside cells.

More than a decade later, the deciphering of endocytic mechanisms involved in CPP uptake as well as the exact receptors used still continues. Recent findings suggest the involvement of a few receptor types in the internalization of CPPs, which prefer CME over other uptake mechanisms.

Kawaguchi et al. [86] have recently reported that syndecan-4, one of the heparan sulfate proteoglycans, is an endogenous membrane-associated receptor for the cellular uptake of R8 peptide via clathrin-mediated endocytosis. RNA interference-mediated knockdown experiments in combination with pharmacological inhibitors support their results. These results contradict the former findings of the group, which stated that syndecans were involved in the uptake of R8 via macropinocytosis. However, there is a possibility for syndecans to have multiple roles in the uptake of CPPs [44].

Indeed, there is a chance that binding to heparan sulfates could provoke several endosomal pathways. Heparan sulfates can be conjugated to a variety of proteins with different spatial distributions such as the cell-surface associated syndecans and glypicans, which further determines the biological outcome of the ligand binding. Syndecans are transmembrane proteins, whereas glypicans are associated with the plasma membrane via a glycosylphosphatidylinositol (GPI) anchor. As a consequence of this contrast, syndecans and glypicans may be preferentially sorted to clathrin-coated and caveolin/lipid raft plasma membrane domains, respectively. Uptake of glypican-bound ligands may proceed primarily through caveolin-dependent endocytosis. This might indicate a possible role for syndecans in clathrin-mediated endocytosis [87].

As an interesting contrast to the common cationic CPPs, Arukuusk et al. [88] suggest negatively charged CPPs as oligonucleotide carriers. The CPPs are not inherently anionic, but confer a negative charge after they are complexed with a nucleic acid in cell culture media. The group has already demonstrated that the uptake of these anionic particles is mediated by the scavenger receptors SCARA3 and SCARA5 [75]. This is logical, since their negative charge would not let them interact with negatively charged components of the plasma membrane, which is the first step in the internalization of nanoparticles in cells, so there must be a receptor involved. Their results have shown that one of their CPP/cargo complexes, the NickFect1 stearylated transportan 10 (TP10) analog, crosses the membrane using clathrin-mediated endocytosis. However, this cannot be regarded as the one and absolute pathway this complex uses, since scavenger receptors are known to be involved in multiple endocytic pathways. Furthermore, this endocytic path may not be used by the peptide alone, and may be the result of cargo attachment to the peptide [77].

#### Caveolae-mediated endocytosis (CvME)

Caveolae are flask-shaped invaginations in the cellular membrane, which have a diameter of about 50–100 nm. They were described for the first time in the early 1950s, as present in many cell types [89]. Caveolae were assumed to be mediators in the transport of serum proteins to tissues across the endothelium of blood vessels. Nowadays, caveolae are known to encircle cholesterol and sphingolipid-rich domains of the plasma membrane, in which a number of signaling molecules may be located [52]. Since they are highly hydrophobic and rich in cholesterol and sphingolipids, some authors often refer to caveolae as lipid rafts [89–90]. Caveolae have been implicated in numerous functions, having important roles in cell signaling, lipid regulation and endocytosis [91].

The most famous ligand for this pathway seems to be albumin. The binding to its receptor, gp60, provokes internalization of the protein. In addition, there is a growing number of receptors, other than the gp60, which are known to induce caveolae-mediated uptake. These receptors are involved in the uptake of ligands such as folic acid, alkaline phosphatase, and pathogens as ganglioside-bound cholera toxin, SV40 virus, polyoma virus, HIV virus. Using caveolae-dependent endocytosis, pathogens can escape the endosomolytic intracellular path.

**Caveolae on the plasma membrane:** The shape and structural organization of caveolae are conferred by members of the caveolin gene family, caveolin-1, -2 and -3 (Cav-1, -2 and -3). Cav-1 and -2 are rather ubiquitous, being highly co-expressed in fibroblasts, adipocytes, endothelial cells and pneumocytes. Cav-3 is expressed independently and is limited to the skeletal musculature and cardiac myocytes. Cav-1 seems to be the one giving shape to the caveole. It is a small integral membrane protein, whose hydrophobic amino acids are inserted into the inner leaflet of the membrane bilayer in a hairpin-like form. The cytosolic region functions as a scaffolding domain and has been implicated in cholesterol and sphingolipid-rich membrane domain binding [52,89,91]. Cav-1 was shown to be highly immobile at the plasma membrane. Therefore, it seems that this protein stabilizes the plasma membrane association of the invaginations, postponing their dynamin-dependent budding and detachment, regulating the constitutive endocytosis. This could also mean that the uptake can be opened by some specific signaling events. If so, ligand internalization via caveolae-dependent endocytosis may be signal mediated in cells expressing Cav-1 [90]. Cav-2 has also been suggested as necessary for the formation of deep plasma membrane-attached caveolae.

Further studies on caveolae-dependent endocytosis have made it clear that caveolae formation requires proteins other than caveolins. The second group of proteins needed is named cavins. In contrast to caveolins, cavins are peripheral membrane proteins which bind molecular components of the caveolar domain facing the cytosol. Recently, a crucial role in caveolae formation has been attributed to PTRF (polymerase I and transcript release factor), also known as cavin-1. In the last steps of caveolae biogenesis, PTRF is recruited to the plasma membrane and it most likely operates as a coat protein for caveolae. Binding of cavin-1 to the domain containing oligomerized caveolins, cholesterol and phosphatidylserine stabilizes the membrane curvature to produce the classical flask-shaped caveolae [89,91].

Two other components which are essential for the caveolae formation are the actin cytoskeleton as well as cholesterol. The actin cross-linking protein filamin is one of the proteins identified as a ligand for Cav-1 and it is thought that PTRF may serve as a direct connection between the caveolae and the cytoskeleton [89].

**Caveolae internalization and trafficking:** Electron microscopy data show that caveolae are tightly connected to the actin filaments, which suggest a role for the cytoskeleton in caveolae-dependent endocytosis. The internalization of caveolae is facilitated by the disruption/reorganization of the cytoskeleton. Local disassembly of the cortical actin network is essential to initiate inward transport of caveolae along microtubules, which serve as transporting tracks. These observations show a dual role for actin in caveolar internalization: one is to keep the organization of caveolae and maintain their immobility at the plasma membrane, and the other is to promote vesicle budding and release from the membrane [91].

Another factor that regulates caveolae budding is the activity of kinases and phosphatases. Cav-1 and -2 are known to be substrates for Src, a tyrosine kinase which phosphorylates the scaffolding domain of both proteins. The simultaneous phosphorylation might equally be important in regulating caveolae budding and pinching off from the cellular membrane. On the contrary, phosphatases seem to inhibit caveolar endocytosis. PP1 and PP2 (protein phosphatases) activity contributes to the dephosphorylation of Cav-1 and -2 [89–90].

Caveolae endocytosis relies heavily on dynamin, a multi-domain GTPase, shown to interact directly with Cav-1. Ligand binding disrupts the local actin cytoskeleton and promotes dynamin II recruitment to the site of internalization. Dynamin oligomerization and subsequent GTP hydrolysis result in the formation of a collar, which constricts the neck of caveolae and results in release from the membrane [91].

The question left after membrane release is whether the internalized caveolae can fuse with endosomes and follow the classical endocytic pathway or there is an alternative pathway involving different cellular compartments. Studies on caveolar internalization have shown that this path is always accompanied by the appearance of grape-like caveolar complexes, which are termed caveosomes. These are pH neutral multi-caveolar structures with a heterogeneous morphology, presumably distinct from the classical endocytic organelles. The future fate of these structures is not entirely clear. Viruses are known to use this pathway to avoid lysosomal degradation. However, other data show that ligands such as the cholera toxin internalized by caveolae can be driven to the classical endocytic organelles [89].

**Caveolae-mediated endocytosis and CPPs:** The first report that caveolae-mediated endocytosis can take part in the uptake of CPPs comes from Fittipladi and co-workers [92]. Their main focus was the internalization of TAT fusion proteins. This observation is a contrast to the aforementioned possible TAT uptake pathways – macropinocytosis and clathrin-mediated endocytosis. However, in this case the specific uptake path might depend mostly on the cargo attached to the peptide.

The research group came to this conclusion after investigating the uptake of TAT fusion proteins in fixed cells as well as in real time using living cells. After using methyl-β-cyclodextrin, they observed an impairment of endocytosis. Furthermore, there was no co-localization observed with transferrin, a common agent used to trace clathrin-mediated endocytosis, while the peptide conjugates clearly co-localized with cholera toxin, a marker for caveolae-mediated endocytosis. In addition, the uptake process seemed to occur slowly, which is incompatible with the fast dynamics described for CME. This might be due to Cav-1, which stabilizes the caveosomes on the cell membrane, and thus, slows down the uptake process [92–93].

It was postulated that this process occurs due to the interaction of the peptide complex with heparan sulfate chains of HSPG. However, based on this idea, one may wonder how the interaction of TAT and HSPG can lead to both CME and CvME. Indeed, the preferred uptake road mainly depends on the association with the proteins conjugated to heparan sulfates (syndecans and glypicans). Glypicans have been shown to prefer CvME for ligand uptake [87]. Fusing the TAT peptide with a protein could favor binding to glypicans, and thus, the uptake via CvME.

Caveolae-mediated endocytosis has also been observed in the uptake of proline-rich CPPs [94]. Proline-rich peptides are a chemically and structurally diverse family of cell-penetrating vectors characterized by the presence of pyrrolidine rings from prolines. The amphipathic group of proline-rich peptides has been particularly effective, demonstrating efficient cellular uptake and no cytotoxicity. Investigations of the uptake pathway of these peptides have shown that their internalization is energy-dependent and they mostly co-localize with cholera toxin. This leads to the conclusion that caveolae-mediated endocytosis is mainly involved in amphipathic, proline-rich CPPs uptake. Furthermore, the uptake is thought to be provoked by the interaction between the CPPs and glycosaminoglycans in the extracellular matrix [94].

Säälik and co-workers [95] have reported that transportan and transportan-10 (TP10) mediate protein delivery using caveolae-mediated endocytosis. Studying the uptake mechanism, the group showed that internalization was impaired by cholesterol depletion and Cav-1 downregulation. Co-localization with markers for caveosomes was also observed. This led to the conclusion that CvME might be one of the paths involved in transportan–cargo complex uptake. However, other mechanisms might have a part as well. What is interesting here is that in both cases, with TAT fusion proteins and transportan–protein complexes, caveolae-mediated uptake seems to be the preferred pathway. This is probably a result of the increase in size due to cargo linkage to the CPP, which is in accordance with the size-dependency of the internalization mechanism. Conjugates larger in size are usually taken up by CvME, while the free peptides might use a different path [96].

Stearylated-transportan analogues were also reported to enter cells via CvME [97]. PepFect14 (PF14) complexes with plasmid DNA (pDNA) were detected in endocytic vesicles close to the plasma membrane. The vesicles were mostly of caveolar origin, clearly distinguishable from clathrin-coated structures. The group describes them as grape-like groups or rosettes. Within 1 h of incubation, PF14/pDNA complexes were found in multivesicular bodies. It is postulated that in this case, CvME is provoked by the interaction of PF14/pDNA complexes with scavenger receptors, since their uptake was significantly decreased after SCARA knockdown.

The following report for CvME involvement in CPP uptake comes from a group working on azurin. Azurin is a 128 amino acid-long copper containing redox protein. As a class, redox proteins are not normally classified as CPPs. However, the amphipathic azurin fragments p18 and p28 containing 54 to 67 amino acids have the α-helical structure of azurin. These fragments represent the protein transduction domains of azurin and are reported to have cell cycle inhibitory and antiangiogenic effects [98–99]. Studying the uptake of these fragments, the group observed that both peptides did not enter cells if cholesterol was depleted, and they highly co-localized with Cav-1 in the first 30 min of incubation. This indicates that, at least in part, internalization was caveolae-mediated.

More recently, the N-terminus of VP1 from chicken anemia virus (CAV), designated as CVP1, was shown to act as CPP and efficiently deliver exogenous molecules through caveolae-mediated endocytosis [100]. While employing several endocytic inhibitors, methylated β-cyclodextrin significantly reduced the uptake of CVP1, which implies caveolae-mediated endocytosis as a possible internalization mechanism. The peptide has shown to be more efficient than TAT in delivering cargo molecules.

#### Clathrin- and caveolae-independent endocytosis

The plasma membrane is the spot of origin for complex endocytic pathways, and we are beginning to get a deeper understanding about the way these pathways are regulated. Having defined a substantial number of molecules involved in CME and CvME, other portals for entry into the cells remain obscure. One such pathway involved specifically in the uptake of lipids and fluids may be clathrin- and caveolae-independent [101].

Nowadays, it is accepted that lipids and lipid–protein interaction have a crucial role in the functional compartmentalization of the plasma membrane into microdomains. The term ‘lipid rafts’ has been used to define these lipid domains, formed by the interaction of sterols and sphingolipids. Lipid rafts are small structures, 40–50 nm in diameter, that diffuse freely on the cell surface. The partitioning of certain macromolecules into lipid rafts eases their internalization via endocytic pathway which is clathrin- and caveolae-independent. Even though caveolae are considered a lipid raft subtype and are sometimes classified as identical structures, it is now well established that endocytosis occurs even in cells devoid of caveolae. This conclusion is supported by the fast kinetics of the independent pathway, knowing that due to Cav-1 caveolae-mediated endocytosis is a slow process. This process was best characterized for cytokine receptors on lymphocytes, glycosylphosphatidylinositol anchored proteins (GPI-AP) and viruses [52,101–102].

Although the mechanisms that govern clathrin- and caveolae-independent endocytosis are still poorly understood, it is known that these coat-free pathways can be dynamin-dependent or -independent. For example, it has been shown that this fluid-phase uptake can occur in the presence of a dynamin mutant form, where the canonical endocytic pathways are blocked [52].

The most famous representative for the dynamin-dependent pathway is the interleukine-2 receptor (IL-2R). Studies confirm that IL-2R subunits associate with lipid raft domains, and dynamin regulates the budding and pinching off from the membrane, while the actin-based machinery facilitates the entry [52]. On the other side, endocytosis in the absence of identifiable coats and a particular pinching machinery posed multiple problems. Recent evidence suggests that, in the absence of a specific coat, lipid accumulation could initiate membrane deformation by physically making the membrane bud and form a vesicle. Endocytosis of GPI-AP does not involve any detectable coat nor is it dynamin-dependent. The GPI-anchor was required for internalization via a pathway sensitive to cholesterol depletion. Immediately after internalization, GPI-AP were seen in labile tubular structures. These structures are termed GEECs (GPI-enriched endosomal compartments). This pathway has been shown to be highly actin-dependent [101,103].

Another dynamin-independent path is the ARF-associated pathway, which is thought to be used by the SV40 virus [102]. This pathway is only one of the mechanisms the virus uses to enter cells, since it is known that infection happens via CvME as well. Studies using the virus have shown that the pathway is cholesterol-dependent, involving coat-free endocytic vesicles with a neutral pH and fast uptake kinetics.

Flotillins appear to outline another, dynamin- and coat-independent pathway, which has been used by GPI-anchored CD59 in HeLa cells. Flotillin 1 and 2 seem to induce membrane invaginations in a dose-dependent manner. Furthermore, phosphorylation by tyrosine kinases seems to activate endocytosis via this pathway. However, some studies suggest that flotillins might be involved also in dynamin-dependent pathways, serving as adaptor proteins for specific cargo.

**CPPs internalized via clathrin- and caveolae-independent endocytosis:** Concerning CPPs, clathrin- and caveolae-independent endocytosis has only been reported in a few cases so far. Azurin and its fragments p18 and p28, which were mentioned earlier as peptides using CvME to enter cells, might also use a caveolae-independent pathway. It is postulated that this process occurs in parallel with CvME [98]. Another case is the internalization of transportan and transportan-10. Although both of the peptides have shown to enter cells via caveosomes, another uptake process is also possible. Flotillins were thought to be involved in the clathrin- and caveolae-independent uptake of transportans [95]. The most recent report of a CPP using this mechanism of internalization comes from Ye and co-workers [104]. The group used low molecular weight protamine (LMWP) as siRNA carrier. It was noted that LMWP/siRNA complexes entered cells in spite of the presence of inhibitors for all the major endocytic pathways as well as GTPase inhibitors. This led to the conclusion that the uptake of the complex might be clathrin- and caveolae-independent and also dynamin-independent, as suggested by the fraction of internalized complexes in the presence of a GTPase inhibitor.

### Release from endosomes

CPPs have proven to be molecules able to hijack or induce one or more endocytic mechanisms. As a result, CPP/cargo complexes tend to accumulate inside endocytic organelles, which more often than not, is not the preferred site of action. However, many reports have now established that CPP/cargo complexes can escape from endocytic organelles and reach the cytosolic space. Therefore, CPPs appear to promote the release of molecules trapped in endocytic vesicles, which is essential for intracellular delivery. As is known, molecules which remain within endosomes cannot display their biological activity. In addition, these molecules are subjected to degradation by acidic pH or hydrolases, as they travel from early endosomes to late endosomes, and finally, are fused with lysosomes [105].

**Mechanisms of endosomal escape and strategies to improve it:** The release from endosomes seems to be a limiting step in the endocytic uptake of CPPs – it determines the efficiency with which a cargo reaches the cytosol. Thus, understanding the mechanisms that underline this process is of great importance. A great part of the challenge in understanding how CPPs escape endosomes lies in the frequent poor efficiency of endosomal release of these systems. This does not pose a problem for cargos which require a small number of copies to elicit a response, however, ones which require a larger number of copies often fail to show biological effects.

**Figure 4:**
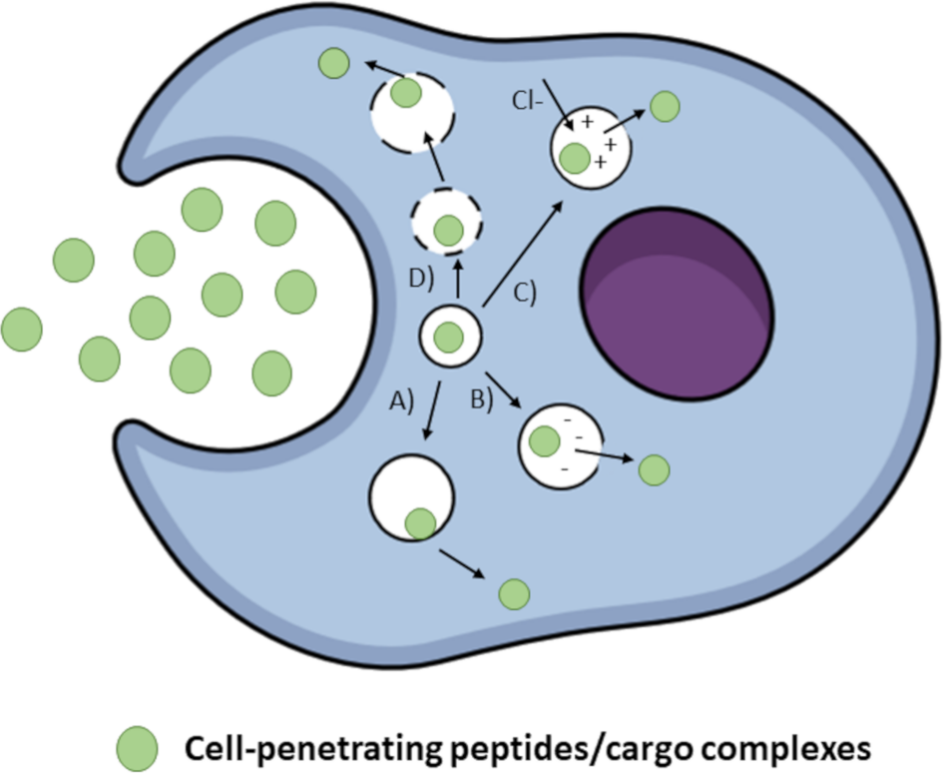
Most commonly used strategies for improving the endosomal release of CPPs. A) Fusogenic lipids, B) pH-sensitive membrane disruptive peptides, C) ‘Proton sponge’ effect, D) Endosomolytic agents such as chloroquine.

Several mechanisms for endosomal escape have been proposed so far. One possible mechanism is based on the ability of CPPs to induce membrane disruption. Positively charged CPPs are thought to interact with negatively charged phospholipids in the endosomal membrane [105]. This interaction would result in the formation of a membrane pore and leakage, which would ease the release of CPPs. TAT has been shown to induce leakage of endosomes after interacting with negatively charged phospholipids in the endosomal membrane [106]. Another possible mechanism for escape is the formation of ionic pairs between CPPs and negatively charged membrane lipids, which would then partition across the endosomal membrane [107]. This mechanism has been proposed for oligo-arginines [108]. In the following, some of the most common strategies used to improve endosomal release are discussed. Figure 4 gives a representation of these mechanisms.

**Strategies to improve endosomal release of CPPs: 1. Use of fusogenic lipids:** Fusogenic lipids have been suggested as a tool to improve the endosomal release of CPPs. The inclusion of a neutral helper lipid such as dioleoylphosphatidylethanolamine (DOPE), is known to greatly enhance the release and activity of the cargo molecule. For instance, DOPE incorporation in lipoplexes or TAT-pDNA complexes showed a vast improvement in transfection efficacy.

The mechanism by which DOPE is thought to mediate endosomal release is the following. At lower pH levels, which are found in endosomes, DOPE shifts its phase from lamellar to an inverted hexagonal phase. The inverted hexagonal phase supports the fusion of the nanocarrier and the endosomal membrane, which finally destabilizes the membrane to release the nanocarrier into the cytosol (Figure 4) [107].

**2. Membrane-disruptive peptides:** What would be a better way of overcoming the endosomal trap than mimicking nature’s mechanisms such as those that viruses use? This can be easily done by conjugating a viral fusion sequence to a nanocarrier. For this purpose, a commonly used peptide is the HA2 peptide. It is a pH-sensitive fusogenic peptide derived from the hemagglutinin protein of influenza virus. The HA2 peptide has an α-helix structure at its N-terminus capable of insertion into membranes. Under the acidic pH conditions in endosomes, a conformational change exposes the α-helix structure, which then fuses with the endosomal membrane and is followed by the release of the virus in the cytosol [23,107]. This method has been used to improve the endosomal release of TAT complexes with proteins, as well as for transportan–peptide nucleic acid (PNA) complexes [60,109].

**3. The ‘proton sponge’ effect:** The ‘proton sponge’ effect, in which the buffering capacity of an agent is used to increase osmotic pressure within endosomes leading to their swelling, rupture and release of contents, has been investigated as another strategy to improve the endosomal release of CPPs. One of the commonly used agents in this case is histidine. At acidic conditions in the endosomes, the imidazole group in histidine is protonated, which results in osmotic swelling and rupture of the endosome [110]. This strategy has been successfully employed to enhance the gene expression of a TAT/pDNA complex [111]. Another way to exploit the proton sponge effect is to use membrane disruptive polymers such as polyethylenimine (PEI). Upon protonation, PEI provokes the rupture of endosomes. PEI has been combined with a TAT/pDNA complex and has improved its cytosolic delivery [107].

**4. Use of endosomolytic agents:** The most famous representative of endosomolytic agents is chloroquine, a weak base that can enter cells and accumulate in endosomes and lysosomes after being protonated. At low concentration, chloroquine can inhibit endosome acidification and thus, prevent its maturation. At high concentration, it can cause endosomal swelling and rupture. Wadia and co-workers [60] reported that chloroquine enhanced the nuclear delivery of TAT fusion proteins. This method has also improved the delivery of siRNA complexed with MPGα [23,84].

**5. Photochemical Internalization:** Photosensitizers that can accumulate in the endosomes have been used to achieve endosomal release. These compounds are able to localize in cell membranes and are taken up by endocytosis, where they again localize in the endosomal membrane. Upon irradiation with light of a specific wavelength, these molecules are able to produce reactive oxygen species (ROS). These ROS will damage and rupture endosomal membranes, causing the release of the endosomal cargo [105].

### Factors affecting the cellular uptake mechanism

The internalization mechanism of CPPs still remains a matter of debate. In spite of many similarities between different CPPs, their uptake mechanisms may vary considerably. This leads to contradictory observations, mostly because there is a great number of factors that affect the cellular uptake and translocation mechanism. In general, the factors that determine the uptake routes of cell penetrating peptides can be divided into two major groups: i) the physicochemical properties, concentration of peptide and its cargo and ii) the properties of the plasma membrane, its lipid and protein composition.

Contradictory results regarding the internalization mechanism of CPPs often arise due to differences in experimental conditions. The fist important factor is the CPP concentration. In many cases, it has been shown that the applied concentration greatly affects the uptake pathway. Another factor is the net charge of the peptides, especially the positive charges coming from arginine residues. Most of the CPPs are rich in arginine residues, and arginine (in particular, its guanidinium group) is more favorable than lysine for the delivery and the uptake of CPP. Amphipathicity is another factor known to influence uptake. Primary and secondary amphipathic peptides can directly penetrate through the cell membrane at low concentration, while nonamphipathic CPPs use endocytosis [1]. The temperature at which the experiment is conducted can also influence the internalization mechanism. Fretz et al. [41] have observed the temperature dependence of R8 translocation across plasma membranes. They have found that at 4 °C diffuse signals from the fluorescently labeled peptide are more prominent in the cytoplasm, which is usually an indicator for a direct translocation across the membrane. However, at 37 °C, both diffuse and punctate signals were observed, pointing to a possible activation of an endocytic mechanism at higher temperature. Cargo molecules attached to CPPs can also greatly influence the uptake path. The roles of cargo, concentration and cell lines will be further discussed below.

#### Role of cargo molecules

The cargo conjugated to the CPP is often a very important parameter of internalization. Tünnemann et al. [112] compared the uptake of TAT conjugated to peptides and globular proteins in living cells. They found that the size of cargo fused to TAT had a great influence on the uptake mechanism. The larger complexes containing proteins were seen inside vesicular structures, while the TAT-peptide conjugates were diffusely distributed throughout the cell. This indicates that the size of the cargo, which naturally influences the size of the overall complex, leads to a different uptake mechanism. The smaller the size, the greater is the chance that the complex will be taken up by direct translocation. At larger dimensions, however, endocytosis prevails. This is similar to the case of the uptake mechanism of unconjugated TAT compared to TAT fused to a cargo, where the presence of a cargo molecule decides between two different endocytosis mechanisms [83,93]. Unconjugated TAT prefers CME to enter cells, while its conjugated counterpart is more likely to use CvME.

The uptake of other arginine-rich peptides is also a subject of cargo influence. Maiolo et al. [113] investigated the effects of cargo molecules on the uptake of R7 and R7W (R7 conjugated to a tryptophan residue). The peptides alone showed diffuse signals in the cytoplasm of the cells used. However, after fusion to cargo peptides, there was a significant reduction in the diffuse signal and a small increase in the punctate signal coming from endocytic vesicles.

#### Role of concentration

The concentration of the CPP is a supreme factor, since it can trigger different uptake pathways. It is believed that endocytosis usually occurs at low peptide concentration, and it switches to direct penetration when the concentration is higher. While investigating the role of temperature in the uptake of R8, Fretz et al. [41] also tested the dependence on the concentration. When lower peptide concentration was used, vesicular labeling was observed in the cytoplasm indicating endocytic uptake. At higher concentration, vesicular and diffuse labeling were present, indicating that endocytosis and direct penetration might occur simultaneously. This was later confirmed by another research group, which observed a similar behavior of the peptide dependent on the concentration [114]. R9 and TAT have also been tested for concentration-dependent uptake [67,114]. These peptides also show predominantly vesicular signals at lower concentration and extensive cytosolic labeling at higher concentration. However, in this case the situation becomes more complicated, because at low concentration (<5 µM) endocytic inhibitors had only a slight effect on the uptake of R9 and TAT, while at higher concentration clathrin inhibitors seem to strongly influence the uptake. Indeed, it was observed that the peptides are, to some extent, taken up by vesicular structures at higher concentration, in addition to the fast, nonendocytic uptake via nucleation zones [67]. As this results suggest, the effect of the CPP concentration on the uptake mechanism can be much more complicated than initially thought.

In contrast, penetratin seems to work differently. For this peptide, it has been shown that direct penetration occurs at low concentration. The switch to endocytosis occurs when the concentration is increased [26].

#### Role of the cell type

The cell lines used in experiments for CPP internalization also have a huge impact on the mechanism of uptake. In particular, the properties of the plasma membrane and the extracellular matrix structure can play a major role in CPP uptake. It is known that the first contact during internalization forms between the positively charged CPPs and the negatively charged GAGs from the extracellular matrix.

Hällbrink et al. [115] investigated how the peptide-to-cell ratio influenced the cellular uptake into Chinese hamster ovary (CHO) cells. To be more specific, they wanted to see how the uptake changes if the number of peptides, instead of the concentration, is increased at a constant cell number. Furthermore, they observed the impact of cell number and confluence on the uptake. What they have shown is that doubling the incubation volume at a fixed number of cells increased the intracellular peptide concentration more efficiently than doubling of the external peptide concentration. Moreover, applying a fixed peptide concentration to different cell densities revealed a decrease of the uptake at higher confluence. This could be caused by the different membrane composition or the different endocytosis behavior of the growing cells. Another possibility is that access to the membrane decreases as confluence increases. These results are an interesting example of how experimental factors in cell culture can influence the uptake efficiency.

Mueller et al. [116] did a profound investigation of the uptake of 22 different CPPs in four different cell lines. They used Cos-7, HEK293, HeLa and MDCK as representative cell lines and studied the internalization of some of the most prominent CPPs such as penetratin, TAT, transportan, Pep-1, MPG, MAP, R7 and R9. The results led to the categorization of CPPs into three groups according to their behavior, showing high (penetratin, transportan, MAP), medium (TAT, Pep-1, MPG) and low cellular uptake. What is interesting here is that the results show cell-dependent uptake for some of the peptides. For example, MPG was preferentially taken up by Cos-7 cells, fibroblast-like cells derived from monkey kidney tissue. This could be due to the fact that MPG has a NLS derived from SV-40 large T antigen, the virus which was used for obtaining the Cos-7 cell line [117–118]. Penetratin was the favorite for HeLa cells, while transportan entered all cell lines with similar efficiency. The effect of endocytosis inhibitors was also shown to be cell-dependent, thus implicating different uptake mechanisms in different cell lines. For example, TAT showed mostly vesicular distribution in all cell types. However, a more diffuse signal, as well as cytoplasmic and nucleic localization was found for HeLa and MDCK cells.

Cell-dependent uptake was also observed for R7 and R7W [113]. Their uptake was investigated using the two different cell lines A431 and U2OS. Endocytic uptake was shown to be more prevalent in A431 than in U2Os cells as measured by the vesicular distribution of the peptides.

It is generally accepted that the first contact that leads to CPP internalization is between the positively charged peptides and the negatively charged GAGs from the extracellular matrix. In this case, heparan sulfate proteoglycans have been given the prominent role of inducing membrane translocation of several CPPs. Although all tissues express proteoglycans, the level of expression is determined by the state of differentiation and growth of the cell, and specific HPSG isoforms are known to be differentially expressed in different cell types [119]. The influence of different HSPG isoforms has yet to be investigated in detail. However, it is known that the uptake of some CPPs depends highly on the presence of these proteoglycans. It has been reported several times that the interaction with HSPGs is essential for the uptake of TAT [83,120]. Proteoglycan-binding has been observed to be important for the internalization of R8 and R9 as well [70,121].

Recently, a study investigated the influence of cell lines on the uptake of a CPP with a potential anticancer activity [122]. The CPP is sC18, a peptide derived from the C-terminal domain of the antimicrobial peptide CAP18. Its uptake was investigated in HeLa, PC-3, HCT-15 and MCF-7 as cancer cell lines, and HEK293 as a noncancer cell line. Interestingly, in all cancer cell lines, a diffuse fluorescent signal next to a punctate distribution was observed in the cytoplasm as well as nuclear accumulation. However, in the noncancer cell line just a punctate distribution was seen, and hardly any peptide in the nucleus was observed.

#### Role of membrane properties

A substantial amount of knowledge has been obtained about the permeability properties of CPPs since they were first discovered. Despite the information about their cell penetration mechanism being sometimes confusing and contradictive, we are familiar with their properties (such as physicochemical properties and concentration) which influence their uptake. However, less is known about the influence of the plasma membrane properties on the uptake. The number of studies on this topic is rather narrow, however, information about the impact of plasma membrane composition and how it changes and adapts is available from studies using poly-arginines [123].

Crosio et al. studied the influence of the membrane properties on the uptake of lysine- and cysteine-modified nona-arginine (KR9C) by utilizing anionic membranes with different fluidity and rigidity (a saturated membrane composed of DOPG/DPPC, an unsaturated membrane composed of DOPG/DOPC or a mixture of DOPC with cholesterol). They found that the peptide adsorbs on the polar regions of all membranes and this leads to the reduction of the anionic membrane charge. The peptide was able to get inserted into both DOPG/DOPC and DOPG/DPPC layers, however, when it came to the monolayers containing cholesterol, its insertion was impaired. This led to the conclusion that cholesterol molecules located in the cell membrane can hinder the uptake of poly-arginines.

The most likely explanation for the insertion of the peptide into the lipid layer is the ability of poly-arginines to recruit negatively charged phospholipid heads and thus decrease the surface pressure of the membrane. The peptide also increased the conductivity of both saturated and unsaturated layers and caused membrane deformations. The membrane deformations are thought to be caused by the charge neutralization, which can lead to a decrease in bending rigidity. A similar behavior was described for the TAT peptide by Herce et al., discussed in more detail in a previous section (Direct translocation mechanisms used by arginine-rich peptides), where the incorporation of water into the membrane together with the peptide as well as membrane thinning have been mentioned [47–48,123].

### In vivo application of CPPs

CPPs demonstrated to be highly efficient in cargo delivery in vitro, being able to mediate the uptake of different cargo molecules in a number of cell lines. However, their success as delivery systems in vivo has been limited, mostly due to in vivo stability issues (they are susceptible to proteolytic degradation), immunogenicity or toxicity due to the lack of specificity [7,124–125]. Up to now, a number of in vivo studies have been done, in which CPPs such as TAT or penetratin have been tested for their cytotoxic and immunogenic potential [3]. The outcome so far shows that CPPs exert low cytotoxicity and no immune response. This gives the promise that, in some cases, these results can be translated to humans [126].

A CPP called PepFect6, which is based on covalently modified TP10 by the introduction of a proton-accepting moiety to facilitate endosomal escape and stearylation for an improved serum stability, was tested for its toxicity and immunogenicity in mice, following systemic administration. The in vivo studies showed that the peptide was mainly distributed in the liver, in the lung or in the kidney, with no associated toxicity and a negligible immune response. Furthermore, more than 60% gene silencing has been observed by using this peptide (PepFect6 was used as a carrier for the HPRT1 house-keeping gene siRNA) [20,124].

Another example of a successful application of a CPP in vivo is given by Toro and co-workers [127–128]. In this case, the TAT protein was used as a protein transduction domain, and it was conjugated to purine nucleoside phosphorylase (PNP), an intracellular enzyme crucial for purine degradation. The toxic and immunogenic effects as well as the effects on the biological activity of the cargo were observed in mice. Defects in PNP activity are known to result in metabolic abnormalities and fatal T cell immunodeficiency. The conjugation of the enzyme to TAT had positive effects on the retention and the distribution of PNP, as well as on the immunogenicity. The TAT-fusion prevented the enzyme from being excreted, while the nonfused PNP was undetectable in blood and tissues only after several hours after administration. Furthermore, TAT-PNP maintained its biological activity and an increase in T cell number was observed, besides an increase in the titer of antibodies against TAT-PNP.

In vivo studies have also been conveyed on Duchenne muscular dystrophy (DMD), a deadly neuromuscular pathology caused by the absence of the dystrophin protein and characterized by a progressive weakness of skeletal muscles. Here, penetratin was used as a CPP conjugated to a NF-κB peptide inhibitor, since NF-κB is known to be a possible target for therapeutic intervention. Mice were treated intraperitoneally and showed an improvement in motor performance. CPPs can also be used in the development of delivery systems for cancer treatment, since oftentimes traditional chemotherapy lacks specificity. For such purpose, the amphipathic peptide MPG has been utilized as a carrier for a siRNA molecule targeting cyclin B1 (a mitosis-regulating protein with altered expression in various forms of cancer). The complexes were functionalized with a cholesterol moiety, in order to be more suitable for systemic administration. After intravenous administration, a significant reduction in the tumor size was observed [126].

An attractive approach in the use of CPPs would be to circumvent the parenteral method of administration and find another less invasive but effective mode of drug delivery system application. Schiroli et al. [129] have reported the use of a peptide for ocular delivery (POD) with cell penetrating properties, known to diffuse into the corneal layers. The peptide was noncovalently complexed to a siRNA molecule, and the knockdown of luciferase reported gene expression in the corneal epithelium was evaluated. After topical administration of the complex, a 30% reduction in the expression was observed, with no inflammatory or toxic effects.

Worth mentioning is an in vivo study done by Ghatnekar et al., who studied the effect of antennapedia cell internalization sequence linked to the C-terminus of connexin43, called ACT1, in wound healing. Mouse and pig models of skin wound healing were used, and wounds were assessed for structural and functional markers of inflammation, scaring and healing. It was shown that the ACT1 peptide promotes regenerative healing and decreases inflammation [130]. As a topically applied peptide, ACT1 also showed promising results in a phase II trial [131]. For further reading on the preclinical and clinical application of CPPs, a great overview is given in a review by Guidotti and colleagues [126].

## Conclusion

CPPs present a major breakthrough as delivery systems for macromolecules. CPPs are capable of entering the body in a noninvasive manner, they do not destroy the integrity of cellular membranes, and are considered highly efficient and safe. Thus far, they have been used to safely deliver molecules such as peptides, proteins and nucleic acids that are generally difficult to deliver due to some of their inherent properties. Therefore, they provide new horizons for research and application in medical sciences.

The uptake of CPPs has been reported in a wide variety of cell types and in combination with different cargo molecules. However, the exact mechanism CPPs use to traverse cell membranes still seems like an unsolvable riddle. As it is stated above, CPPs use different mechanisms to enter cells. In general, the mechanisms can be divided into two broad groups: endocytosis and direct penetration. Nevertheless, there is not even one CPP that strictly falls into one of the two categories. In the literature, it is described that many CPPs use different uptake pathways depending on their structure, net charge, concentration, type of cargo, cell lines used, temperature at which uptake studies were conducted and incubation times. The high variability in these factors between different laboratories leads to controversial results regarding the internalization mechanism, so at some points, this obstacle seems very hard to overcome. Undoubtedly, by further investigating the influence of each parameter and by the standardization of the recent experimental methods, more light will be shed on the mechanisms CPPs use to enter cells. Or, to freely quote Richard Feynman – “There’s plenty of room at the bottom”.
